# Evaluation of treatment outcomes among adult patients diagnosed with tuberculosis in Ghana: A 10 year retrospective review

**DOI:** 10.1016/j.ijregi.2023.11.004

**Published:** 2023-11-10

**Authors:** Peter Puplampu, Isaac Kyeremateng, Olive Asafu-Adjaye, Anita Ago Asare, Kofi Agyabeng, Roderick Sarkodee, Oladele Oluwakemi, Vincent Ganu

**Affiliations:** 1 Department of Medicine & Therapeutics, University of Ghana Medical School, Accra, Ghana; 2 Infectious disease unit, Department of Medicine, Korle Bu Teaching Hospital, Accra, Ghana; 3 Ghana Health Service, Accra, Ghana; 4 Greater Accra Regional Hospital, Accra, Ghana; 5 Department of Community Health, University of Ghana Medical School, Accra, Ghana; 6 Department of Biostatistics, School of Public Health, University of Ghana, Accra, Ghana

**Keywords:** Tuberculosis, Outcome, Extra-pulmonary, Clinically diagnosed, Treatment success rate

## Abstract

**Objectives:**

The study determined tuberculosis (TB) treatment outcomes in Southern Ghana from 2012 to 2021.

**Methods:**

A retrospective analysis of service data on TB cases was conducted. Treatment success was defined as TB cure or completion of treatment course, whereas unsuccessful outcomes was defined as death, failure of treatment and lost to follow up. Bivariate and multivariate logistic regression was used to determine factors associated with treatment outcomes.

**Results:**

A total of 4106 adult TB cases were reported with a median age of 41 (interquartile range 32-52) years. Of these, 93.1% (n = 3823) were newly diagnosed. The treatment success rate declined from 71.0% in 2012 to 55.7% in 2021 (ktau-b = −0.56, *P* = 0.0318). Clinically diagnosed TB and extra-pulmonary TB had 7.0% (adjusted prevalence ratio [aPR]: 0.93, 95% confidence interval [CI]: 0.88-1.00) and 24.0% (aPR: 0.76, 95% CI: 0.69-0.84) respectively, less successful treatment outcome compared to pulmonary TB patients. HIV negative status was associated with 22% higher successful treatment outcome compared with being HIV positive (aPR: 1.22, 95% CI: 1.12-1.33).

**Conclusion:**

Tuberculosis treatment success rate declined over the period. There is a need for the TB Control Programme to review the national and sub-national TB data to ascertain poor performing TB treatment sites to identify and address context specific challenges with treatment interventions and system inadequacies to improve treatment success rates.

## Highlights

• There has been general decline in success rates of tuberculosis (TB) treatment from 2012 to 2021.
• TB treatment completion rates are higher than TB cure rates.
• Increased odds of successful treatment outcomes among HIV negative patients.
• Extra-pulmonary TB and age ≥65 had reduced odds of successful treatment outcomes.

## Introduction

An estimated 10.6 million people were affected with tuberculosis (TB) worldwide with a total of 1.6 million people (including 214,000 with HIV) dying of this infectious disease in 2021 [1]. The majority of persons with TB live in Asia and Africa [1]. The 2021 Global TB report indicates a decline in the number of TB cases reported with an increase in TB-related deaths at all levels, falling short of the targets set in the End TB strategy 2015-2035. The End TB targets for 2035 are to reduce the TB incidence rate by 90% to ≤10 cases per 100,000 population per year and to reduce the absolute number of TB deaths by 95% compared with a baseline of 2015 [1]. Successful treatment of TB is necessary in reducing deaths and other complications especially in high burden countries across Asia and Africa.

Globally, though treatment success rates (TSR) have remained relatively stable across all ages, TSR has still fallen short of the 90% rate recommended by the World Health Organization (WHO) [1], [2]. TSR also remain lower in people living with HIV (PLHIV) though some progress have been made overtime [1]. Treatment success rates reported for Sub-Saharan Africa range from 69-94% [1]. Proposed predictors of successful TB treatment reported include the female sex, HIV-negative status and being of a young age [3], [4], [5], [6], [7]. HIV infection, socioeconomic factors, adverse reactions from TB drugs, undernutrition, alcohol abuse and old age have been noted to be risk factors for poor outcomes in persons with TB infection [5], [8], [9].

Ghana is a country with a high burden TB/HIV with TB being ranked as the seventh top cause of mortality in Ghana accounting for 4.9% of all deaths in 2019 [10], [11]. The prevalence of TB incidence by microscopy was 111/100,000 and that of bacteriologically confirmed TB was 356/100,000 in 2013 [12]. The TB case detection rate in Ghana is 34% which is very low compared to the WHO set target of 80% [13], [14], [15]. With a low rate of TB case detection, it is important to ensure identified TB patients complete treatment and are cured. Evaluation of TB treatment outcomes is key to ensure that patients do not go back into communities to infect others and also reduce the risk of developing drug resistant TB.

The WHO recommends that all countries monitor their progress using the high-level indicators/targets set in the End TB strategy 2015-2035 [1]. This study sought to determine TB treatment outcomes over a 10-year period, 2012 to 2021 in southern Ghana.

## Methods

### Study design and settings

A facility-based retrospective study of TB cases treated from January 2012 to December 2021 was conducted at the TB unit of the Korle-Bu Teaching Hospital (KBTH) in Accra Ghana. The TB unit serves as the main referral center for the management of TB in Southern Ghana. It offers both in- and out-patient services.

### Study participants

Study participants were patients 18 years and above with clinical and microbiological diagnosis of drug-sensitive TB who were registered and initiated on anti-TB treatment between January 2012 to December 2021.

### Data source and extraction

Data were extracted from the TB register using a standardized data extraction form that included information on socio-demographic variables, medication-related factors, and treatment result.

### Outcome measures and definitions

Definitions of outcome measures for this study were as per WHO TB framework [16]. The TB treatment outcome measures and their definitions are as below:

***Cured***: A pulmonary TB patient with bacteriologically confirmed TB at the beginning of treatment who was smear- or culture-negative in the last month of treatment and on at least one previous occasion.
***Completed***: A TB patient who completed treatment without evidence of failure BUT with no record to show that sputum smear or culture results in the last month of treatment and on at least one previous occasion were negative, either because tests were not done or because results are unavailable.
***Treatment success***: The sum of cured and treatment completed.
***Failure:*** A TB patient whose sputum smear or culture is positive at month 5 or later during treatment.
***Death:*** A TB patient who dies for any reason before starting or during the course of treatment.
***Lost to follow-up:*** A TB patient who did not start treatment or whose treatment was interrupted for 2 consecutive months or more.
***Not evaluated:*** A TB patient for whom no treatment outcome is assigned. This includes cases “transferred out” to another treatment unit as well as cases for whom the treatment outcome is unknown to the reporting unit.

### Data handling and analysis

Extracted data were entered into Microsoft Excel spreadsheet for cleaning and coding then imported into Stata version 16 for analysis. Summary statistics of categorical characteristics of patients were reported as frequencies and percentages while the median and interquartile ranges were reported for the continuous variables that were not normally distributed. Chi-square test of independence was used to test for association between categorical independent variables and TB treatment outcome. Line graph was used in exhibiting the distribution of TB cases treated and treatment outcomes over time. Mann-Kendall test was used in testing for the significance of trends in the treatment outcome of the study period. The treatment result was classified into two groups: Successful treatment outcomes (which comprised of “Cured” and “Treatment completed”.) and unsuccessful treatment outcomes (“treatment failure”, “died” and “lost to follow up”). Modified Poisson regression model with robust standard error was used in quantifying the effect of between successful TB treatment outcomes and the patients characteristics on successful TB treatment outcomes. Binary logistic regression models with robust standard errors was used as sensitivity analysis. All statistical analysis were done at 5% level of significance.

## Results

Patients treated for TB at the TB Unit from 2012 to 2021 were a total of 4623. Of this, 4453 (96.3%) were aged 18 years and above were included in the study. Out of the 4453, 196 were transferred out of the TB unit so were excluded and an additional 151 were excluded due to missing or repeated entries.

A total of 4106 patients with TB were included in this study. Study participants were aged 18 years to 110 years with a median age of 41 (interquartile range 32-52) years (Table 1). Most of the patients were males (63.4%, n = 2605). More than half (52.6%, n = 2159) of the TB cases were pulmonary TB (PTB) cases (bacteriologically diagnosed (PTB+) - 34.6% (n = 1420) and clinically diagnosed TB 18% (n = 739)) and 47.2% (n = 1940) of them were extrapulmonary TB (EPTB) cases (Table 1). About nine in every ten sampled cases studied were newly diagnosed 93.0% (n = 3823).

**Table 1:** Sociodemographic and clinical characteristics of the notified TB cases (n = 4106) among adult patients at the Chest Unit of the Korle-Bu Teaching Hospital from 2012 to 2021.

Characteristics	2012 N(%)	2013 N(%)	2014 N(%)	2015 N (%)	2016 N (%)	2017 N (%)	2018 N (%)	2019 N (%)	2020 N (%)	2021 N (%)	Total N (%)
All cases of TB treated	514	267	560	433	469	465	378	375	358	287	4106
Age group											
18-34	183(35.6)	86(32.2)	192(34.3)	117(27.0)	128(27.3)	162(34.8)	117(31.0)	116(30.9)	118(33.0)	91(31.7)	1310(31.9)
35-64	295(57.4)	160(59.9)	329(58.8)	275(63.5)	289(61.6)	257(55.3)	237(62.7)	222(59.2)	207(57.8)	168(58.5)	2439(59.4)
65+	36(7.0)	21(7.9)	39(7.0)	41(9.5)	52(11.1)	46(9.9)	24(6.3)	37(9.9)	33(9.2)	28(9.8)	357(8.7)
Median age (Lower quartile – upper quartile)	**40(31-52)**	**41(32-51)**	**40(32-50)**	**42(34-53)**	**42(34-53)**	**42(30-53)**	**42.5(32-52)**	**42(32-54)**	**41(31-51)**	**44(31-55)**	**41(32-52)**
Sex											
Male	322(62.6)	165(61.8)	347(62.0)	284(65.6)	302(64.4)	292(62.8)	245(64.8)	218(58.1)	241(67.3)	189(65.9)	2605(63.4)
Female	192(37.4)	102(38.2)	213(38.0)	149(34.4)	167(35.6)	173(37.2)	133(35.2)	157(41.9)	117(32.7)	98(34.1)	1501(36.6)
Diagnostic classification											
Pulmonary TB+	175(34.0)	99(37.1)	179(32.0)	138(31.9)	140(29.9)	129(27.7)	105(27.8)	151(40.3)	159(44.4)	145(50.5)	1420(34.6)
Clinically diagnosed TB	128(24.9)	66(24.7)	122(21.8)	84(19.4)	94(20.0)	72(15.5)	54(14.3)	46(12.3)	37(10.3)	36(12.5)	739(18.0)
Extra pulmonary TB	210(40.9)	102(38.2)	259(46.3)	211(48.7)	232(49.5)	264(56.8)	219(57.9)	178(47.5)	162(45.3)	103(35.9)	1940(47.2)
Not documented	1(0.2)	-	-	-	3(0.6)	-	-	-	-	3(1.0)	7(0.2)
Type of patient											
New	469 (91.2)	247(92.5)	517 (92.3)	405(93.5)	444(94.7)	444(95.5)	357(94.4)	354(94.4)	335(93.6)	251(87.5)	3823 (93.1)
Other	45 (8.8)	20 (7.5)	43 (7.7)	28 (6.5)	25 (5.3)	21 (4.5)	21 (5.6)	21 (5.6)	23 (6.4)	36 (12.5)	283 (6.9)
HIV status											
Negative	271 (52.7)	134 (50.2)	284 (50.7)	239 (55.2)	225 (48.0)	257 (55.3)	229 (60.6)	196 (52.3)	201 (56.1)	172 (59.9)	2208 (53.8)
Positive	100 (19.5)	47(17.6)	74 (13.2)	59 (13.6)	89 (19.0)	77 (16.6)	69 (18.3)	88 (23.5)	55 (15.4)	41 (14.3)	699 (17.0)
Not documented	143 (27.8)	86 (32.2)	202 (36.1)	135 (31.2)	155 (33.0)	131 (28.2)	80 (21.2)	91 (24.3)	102 (28.5)	74 (25.8)	1199 (29.2)
Chest x-ray findings											
Suggestive	444 (86.4)	243(91.0)	498 (88.9)	380(87.8)	322(68.7)	365(78.5)	315(83.3)	306(81.6)	279(77.9)	172(59.9)	3324 (81.0)
Not suggestive	46 (8.9)	11 (4.1)	39 (7.0)	15 (3.5)	103(22.0)	34 (7.3)	20 (5.3)	33 (8.8)	30 (8.4)	17 (5.9)	348 (8.5)
Unknown	1 (0.2)	1 (0.4)	1 (0.2)	-	1 (0.2)	2 (0.4)	-	1 (0.3)	-	-	7 (0.2)
Not documented	23 (4.5)	12 (4.5)	22 (3.9)	38 (8.8)	43 (9.2)	64 (13.8)	43 (11.4)	35 (9.3)	49 (13.7)	98 (34.1)	427 (10.4)
Treatment outcomes											
Completed	303(58.9)	126 (47.2)	235 (42.0)	176 (40.6)	199 (42.4)	208 (44.7)	182 (48.1)	170 (45.3)	151 (42.2)	134 (46.7)	1884 (45.9)
Cured	62 (12.1)	49 (18.4)	81 (14.5)	52 (12.0)	49 (10.4)	60 (12.9)	37 (9.8)	39 (10.4)	38 (10.6)	26 (9.1)	493 (12.0)
Died	134 (26.1)	84 (31.5)	185(33.0)	172(39.7)	179(38.2)	157(33.8)	119(31.5)	105(28.0)	118(33.0)	83(28.9)	1336 (32.5)
Failure	9 (1.8)	4 (1.5)	8 (1.4)	5 (1.2)	3 (0.6)	3 (0.6)	1 (0.3)	3 (0.8)	2 (0.6)	3 (1.0)	41 (1.0)
Lost to follow-up	6 (1.2)	4 (1.5)	51 (9.1)	28 (6.5)	39 (8.3)	37 (8.0)	39 (10.3)	58 (15.5)	49 (13.7)	41 (14.3)	352 (8.6)
Overall treatment											
Successful	365 (71.0)	175(65.5)	316 (56.4)	228(52.7)	248(52.9)	268(57.6)	219(57.9)	209(55.7)	189(52.8)	160(55.7)	2377 (57.9)
Unsuccessful	149 (29.0)	92 (34.5)	244 (43.6)	205(47.3)	221(47.1)	197(42.4)	159(42.1)	166(44.3)	169(47.2)	127(44.3)	1729 (42.1)

TB, tuberculosis; %; column %

From the chest x-ray findings, 81.0% (n = 3324) of cases were suggestive of TB. For 29.2% (n = 1199) of all TB cases, more than half of the patients were HIV negative (53.8%, n = 2208).

**Figure 1:**
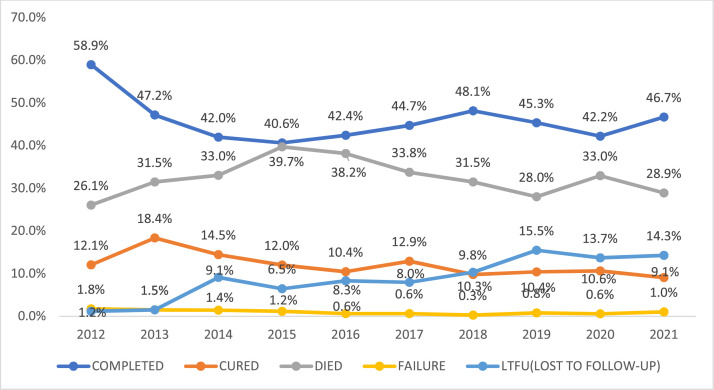
Trend in tuberculosis treatment outcomes among adult patients at the Chest Unit of the Korle-Bu Teaching Hospital from 2012 to 2021.

Majority (57.9%, n = 2877) of the treated patients had successful treatment outcome (completed and cured) (Table 1 and Figure 1).

### Overall trend in treatment success rates

**Figure 2:**
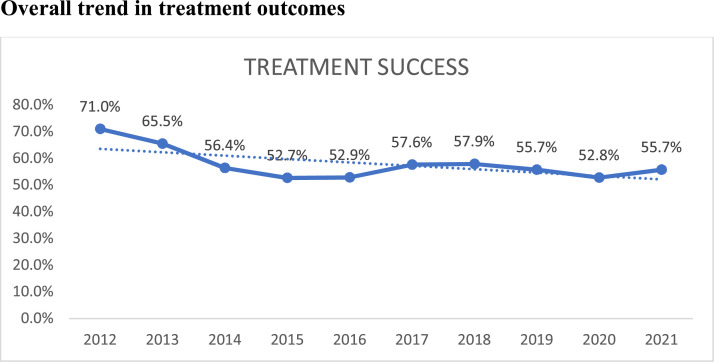
Trend of proportion of successful treatment outcomes among the adult patients at the Chest Unit of the Korle-Bu Teaching Hospital from 2012 to 2021.

From Table 1, the overall TSR averaged 57.9% over the 10-year period. The TSR declined significantly from 71.0% in 2012 to 55.7% in 2021 (ktau-b = −0.56, *P*-value=0.0318) (Figure 2).

### Trend in tuberculosis treatment success rates by HIV status

Although there was a generally decreasing trend in the proportion of successful treatment outcomes among the patients, HIV negative patients recorded consistently higher successful treatment outcomes than HIV positive patients (Table 2).

**Table 2:** Treatment outcomes by sociodemographic and clinical characteristics among adult patients at the Chest Unit of the Korle-Bu Teaching Hospital from 2012 to 2021.

Sociodemographic and clinical characteristics	Treatment outcomes					
	Completed n(%)	Cured n(%)	Died n(%)	Failure n(%)	Lost to follow-up n(%)	*P*-value
**Age groups**						
18-34 years	665(50.8)	194(14.8)	307(23.4)	17(1.3)	127(9.7)	
35-64 years	1082(44.4)	277(11.4)	871(35.7)	20(0.8)	189(7.7)	<0.001
65+ years	137(38.4)	22(6.2)	158(44.3)	4(1.1)	36(10.1)	
**Sex**						
Male	1180(45.3)	355(13.6)	828(31.8)	29(1.1)	213(8.2)	0.001
Female	704(46.9)	138(9.2)	508(33.8)	12(0.8)	139(9.3)	
**Diagnostic classification**						
Pulmonary TB+	501(35.3)	477(33.6)	305(21.5)	36(2.5)	101(7.1)	
Clinically diagnosed TB	443(59.9)	7(0.9)	217(29.4)	2(0.3)	70(9.5)	<0.001
Extra pulmonary TB	936(48.2)	8(0.4)	812(41.9)	3(0.2)	181(9.3)	
**HIV status**						
Negative	1058(47.9)	390(17.7)	528(23.9)	31(1.4)	201(9.1)	
Positive	326(46.6)	49(7.0)	269(38.5)	5(0.7)	50(7.2)	<0.001
**Chest x-ray findings**						
Suggestive	1530(46.0)	449(13.5)	1032(31.0)	37(1.1)	276(8.3)	
Not suggestive	171(49.1)	2(0.6)	135(38.8)	-	40(11.5)	<0.001
Unknown	3(42.9)	1(14.3)	2(28.6)	-	1(14.3)	

TB, tuberculosis; %; Row percentage.

### Tuberculosis treatment outcomes by sociodemographic and clinical characteristics

The proportion of successful treatment outcome decreased significantly with increase in age (*P*-value < 0.001) (Table 3). Among the males, 45.3% (n = 1180) completed the treatment, 31.8% (n = 828) died, 13.6% (n = 355) were cured, 8.2% (n = 213) lost to follow-up, and 1.1% (n = 29) had treatment failure. Among the patients who tested HIV +, 46.6% (n = 326) completed the treatment, 7.0% (n = 49) were cured 38.5% (n = 269) died, 7.2% (n = 50) were lost to follow-up and 2.5% (n = 36) had treatment failure.

**Table 3:** Factors associated with the overall treatment outcomes among adult patients at the Chest Unit of the Korle-Bu Teaching Hospital from 2012 to 2021.

	Treatment outcome			Unadjusted modified Poisson regression model		Adjusted modified Poisson regression model		Adjusted binary logistic regression model	
Characteristics	Successful	Unsuccessful	*P*-value						
	n (%)	n (%)		Unadjusted PR (95% CI)	*P*-value	Adjusted PR (95% CI)	*P*-value	Adjusted odds ratio (95% CI)	*P*-value
**Age group**			**<0.001**		<0.001		<0.001		<0.001
18-34	859 (65.6)	451(34.4)		1		1		1	
35-64	1359(55.7)	1080(44.3)		0.85(0.81-0.90)	<0.001	0.87(0.83-0.92)	<0.001	0.69(0.58-0.82)	<0.001
65+	159(44.5)	198(55.5)		0.69(0.61-0.78)	<0.001	0.68(0.59-0.79)	<0.001	0.40(0.30-0.55)	<0.001
**Sex**			0.077		0.074		0.834		0.857
Male	1535(58.9)	1070(41.1)		1		1		1	
Female	842(56.1)	659(43.9)		0.95(0.90-1.00)		1.00(0.96-1.04)		0.99(0.89-1.10)	
**Type of patient**			0.012		0.004		0.364		0.330
New	2193(57.4)	1630(42.6)		1		1		1	
Other	184(65.0)	99(35.0)		1.13(1.04-1.23)		1.02(0.98-1.06)		1.07(0.93-1.22)	
**Diagnostic classification**			**<0.001**		<0.001		<0.001		<0.001
Pulmonary TB+	978(68.9)	442(31.1)		1		1		1	
Clinically diagnosed TB	450(60.9)	289(39.1)		0.89(0.83-0.95)		0.93(0.88-1.00)	0.040	0.80(0.65-0.98)	0.027
Extra pulmonary TB	944(48.7)	996(51.3)		0.71(0.67-0.75)	<0.001	0.76(0.69-0.84)	<0.001	0.50(0.39-0.62)	<0.001
Not documented	5(71.4)	2(28.6)		1.04(0.65-1.66)	0.878	1.12(0.96-1.30)	0.150	1.29(0.81-2.04)	0.284
**HIV status**			**<0.001**		<0.001		<0.001		<0.001
Positive	375(65.6)	324(34.4)		1		1		1	
Negative	1448(53.6)	760(46.4)		1.22(1.13-1.31)	<0.001	1.22(1.12-1.33)	<0.001	1.67(1.36-2.04)	<0.001
Not documented	554(46.2)	645(53.8)		0.85(0.78-0.93)	0.001	0.91(0.80-1.04)	0.154	0.85(0.65-1.10)	0.221
**Chest x-ray finding**			**<0.001**		**0.001**		0.528		0.456
Suggestive	1979(59.5)	1345(42.9)		1		1		1	
Not suggestive	173(49.7)	175(50.3)		0.84(0.76-0.94)	0.002	0.97(0.80-1.18)	0.759	0.93(0.63-1.38)	0.708
Unknown/Atypical	4(57.1)	3(42.9)		0.96(0.51-1.83)	0.904	1.04(0.56-1.93)	0.899	1.12(0.24-5.16)	0.881
Not documented	221(51.8)	206(48.2)		0.88(0.80-0.97)	0.007	0.91(0.80-1.03)	0.138	0.79(0.59-1.05)	0.108

%, Row percentage; CI, confidence interval; PR, prevalence ratio; TB, tuberculosis.

### Factors associated with treatment outcomes

From both the adjusted modified Poisson and binary logistic regression models, age, diagnostic classification, and HIV status were significantly associated the successful patients treatment outcome (*P*-value < 0.05). The prevalence/ proportion of patients with successful treatment outcome were 13.0% (adjusted prevalence ratio [aPR]: 0.87, 95% confidence interval [CI]: 0.83-0.92), and 32.0% (aPR: 0.68, 95% CI: 0.59-0.79) lower among patients aged 35-64 years and 65+ years compared to patients aged 18-34 years old respectively (Table 3). HIV negative status was associated with 22.0% higher proportion of successful treatment outcome compared with being HIV positive (aPR: 1.22, 95% CI: 1.12-1.33). Clinically diagnosed TB and EPTB patients, had 7.0% (aPR: 0.93, 95% CI: 0.88-1.00) and 24.0% (aPR: 0.76, 95% CI: 0.69-0.84) less proportion of successful treatment outcome compared to PTB+ patients respectively (Table 3).

## Discussion

This study determined the TB trends and treatment outcomes of drug sensitive TB in a tertiary health facility. About 58% had successful treatment. The TSR averaged 57.9% over the 10-year period, declining significantly from 71.0% in 2012 to 55.7% in 2021. HIV-negative patients recorded consistently higher successful treatment outcomes than HIV-positive patients. Being HIV-negative was associated with 65% higher odds of successful treatment outcome compared to being HIV-positive.

Despite concerted efforts being made by the country with its well-established community-based DOTS strategy, TB case reports still do not meet targets set by the WHO. The national TB programm suggested that the drop in case notifications between 2012-2013 was possibly due to underdiagnosis within the routine system [17]. The drop within the period of 2020-2021 could likely be due to many causes chief among them would be a reduction in testing due to the COVID-19 pandemic as seen globally [1], [9]. In addition to this, more male cases were notified globally [1]. Comparable studies across Africa reported a similar significant reduction in TB case notifications with more notifications in men with a few reporting a higher burden in women and children [10], [11]. Despite this, as the country strives to improve TB services being offered, it is important that the excess burden experienced by men be taken into consideration [18].

It has been shown by various studies done within the country that co-infection with HIV negatively impacts treatment outcomes of TB cases [12], [13], [14]. Despite this a significant number of cases in our study have unknown HIV status. This informs that further efforts must be made to improve HIV testing among persons diagnosed with TB.

The TSR declined significantly over the past decade in this study. The TSR of TB patients over the period of 57.9% is much lower than the 90% standard set by the World Health Organization in 2003 and also much lower than TSR recorded in other parts of the country, including 82.5%- 88.1% [15], [16], [17]. It is also lower than that found in Ethiopia of 75.2-88% [19]. However a study done from 2007 to 2017 in the Ashanti Region of Ghana showed a TSR of 68.4% [20]. Unfortunately, the low TSR has showed a general downward trend in our study over the time period. Successful treatment outcome rates fell from 2019 (55.6%) into 2020 (52.9%) at the height of COVID- 19, with rates rising again in 2021 (56.3%) as was found generally globally that COVID- 19 had reversed some gains in TB treatment indicators [1]. Decline in TSR has been attributed to co-infection with HIV, increase in age, in addition to possibility of multidrug resistant TB and the potential for drug-drug interactions in situations of HIV co-infection [21]. Commensurate with the decline in TSR was an increase in the number of deaths within the period 2019-2021; so was the trend globally during the COVID- 19 pandemic [1].

Although it is desirable for cure rates to be higher than completed rates (90% and 85% consecutively by, our cure rates were lower than the completed rates [22]. This could be due to the testing capacity of the Korle-Bu Teaching Hospital being low due to logistics being relatively unavailable or the skills of the laboratory staff being low. Therefore, there is a failure to confirm bacteriological cure for completed treatment outcomes.

Our study found that patients aged between 35-64 and 65+ were less likely to have successful treatment outcomes as compared to those between the ages of 18-34 years. This was similar to other studies. In a study done among patients in a refugee camp in Ethiopia, patients aged above 45 years were likely to have unsuccessful outcomes [19]. This was attributed to the high tendency of poor compliance to drugs. Similarly in a study done in the greater Accra Regional Hospital it was observed that patients above 64 years were more likely to have unsuccessful treatment outcomes [20]. This was associated with the poor immune system from aging.

Patients who were clinically diagnosed (smear negative PTB-) and those with EPTB were also less likely to experience successful outcomes as compared to patients diagnosed of PTB+. This finding is congruent with that found by during a research in Ghana [13]. In their study PTB+ patients were 0.66 times less likely to have unsuccessful outcomes as compared to patients who were clinically diagnosed and those with EPTB. Similarly other studies discovered that the risk of adverse outcomes increased among smear negative and EPTB patients, principally attributed to delays in diagnosis [23].

It was found that HIV negative patients had increased odds of successful treatment outcomes as compared to TB patients who were HIV positive. This is line with several other studies’ findings [24], [25]. These studies reported increased odds of unsuccessful outcomes among TB/HIV coinfection patients. Factors for the poor outcomes are drug-drug interaction, increased comorbidities, and lack of cotrimoxazole prophylaxis therapy [13].

Our study was not without limitations. Firstly, the study lacked the inclusion of some significant factors like education status, family size, residence, family support and medication side effects which have been reported to be associated with TB treatment outcomes [26]. The limitation experienced was mainly due to poor data completeness. However, data quality standards were ensured by training data collectors and supervisors. Secondly the study encountered limitations with missing data on some variables like weight, HIV testing, ART initiation and drug adherence. These variables could not be analyzed.

## Conclusion

The study reported decline in TB case notifications with a significant drop in TSR over the 10-year period with the country being unable to meet the TB targets and milestones especially in area of treatment outcomes. The decline in TSR also occurred at the same time of the occurrence of the COVID-19 pandemic.

## Declarations of Competing Interest

The authors have no competing interests to declare
